# Omega-3 Index and Clinical Outcomes of Severe COVID-19: Preliminary Results of a Cross-Sectional Study

**DOI:** 10.3390/ijerph18157722

**Published:** 2021-07-21

**Authors:** Rodrigo Zapata B., José Miguel Müller, Juan Enrique Vásquez, Franco Ravera, Gustavo Lago, Eduardo Cañón, Daniella Castañeda, Madelaine Pradenas, Muriel Ramírez-Santana

**Affiliations:** 1 Faculty of Medical Science, Universidad de Santiago de Chile and Neurosurgery Service, Hospital Regional Libertador Bernardo O’Higgins, Rancagua 2820000, Chile; rzapata.barra@gmail.com (R.Z.B.); jmmullerr@gmail.com (J.M.M.); kikevas@hotmail.com (J.E.V.); fraveraz@gmail.com (F.R.); 2 Hospital Clínico Fusat, Rancagua 2820000, Chile; drlagogus@gmail.com; 3 Hospital Regional Libertador Bernardo O’Higgins, Rancagua 2820000, Chile; eduardo.canonaedo@gmail.com (E.C.); elsadaniella@gmail.com (D.C.); mpradenasramirez@gmail.com (M.P.); 4 Public Health Department, Faculty of Medicine, Universidad Católica del Norte, Coquimbo 1780000, Chile

**Keywords:** Omega-3 fatty acids, COVID-19, Omega-3 Index, inflammation

## Abstract

The potentially detrimental effects of the worldwide deficiency of Omega-3 fatty acids on the COVID-19 pandemic have been underestimated. The Omega-3 Index (O3I), clinical variables, biometric indices, and nutritional information were directly determined for 74 patients with severe COVID-19 and 10 healthy quality-control subjects. The relationships between the OI3 and mechanical ventilation (MV) and death were analyzed. Results: Patients with COVID-19 exhibited low O3I (mean: 4.15%; range: 3.06–6.14%)—consistent with insufficient fish and Omega-3 supplement consumption, and markedly lower than the healthy control subjects (mean: 7.84%; range: 4.65–10.71%). Inverse associations were observed between O3I and MV (OR = 0.459; C.I.: 0.211–0.997) and death (OR = 0.28; C.I.: 0.08–0.985) in severe COVID-19, even after adjusting for sex, age, and well-known risk factors. Conclusion: We present preliminary evidence to support the hypothesis that the risk of severe COVID-19 can be stratified by the O3I quartile. Further investigations are needed to assess the value of the O3I as a blood marker for COVID-19.

## 1. Introduction

The more severe spectrum of Coronavirus disease 2019 (COVID-19) represents an unprecedented challenge to modern public health and critical care medicine worldwide. An age of 65 years or above and comorbidities, such as chronic cardiovascular disease (CVD) and obesity, have been increasingly associated with severe COVID-19 [1,2,3,4]. Accumulating evidence strongly suggests inflammation-induced lung injury and dysregulated immune responses play crucial roles in the pathophysiology of severe COVID-19 [5,6]. Resolution of acute inflammation has been progressively recognized as an active biochemical process that is necessary for timely recovery of tissue homeostasis after injury [7,8,9]; however, the factors that determine unresolved inflammation in COVID-19 have been poorly investigated. The unresolved inflammation in COVID-19 could largely be determined by deficiency in the long-chain Omega-3 fatty acids eicosapentaenoic acid (EPA) and docosahexaenoic acid (DHA) [10]; this scenario represents one of the most extended micronutrient deficiencies worldwide and is particularly severe in Western countries [11,12]. EPA and DHA are the main precursors of a novel superfamily of autacoids, now termed specialized pro-resolving mediators (SPMs), that are critically involved in the homeostasis of inflammation resolution and airway mucosal immunity [10,13,14,15]. Interestingly, the populations of Japan and South Korea have high blood levels of EPA and DHA, and both countries have reported an extremely low severity of COVID-19 [16,17].

Identification of a reliable blood marker for COVID-19 risk assessment would provide valuable information to assist healthcare decisions by stratifying the risk of individual patients and estimating population vulnerability. The Omega-3 Index (O3I)—the most reliable surrogate marker of Omega-3 status—is the red blood cell (RBC) EPA and DHA content expressed as a percentage of the total weight of RBC membrane fatty acids [18,19]. Although long-term intake of Omega-3 fatty acids is the main predictor of the O3I, other factors are positively (+) or negatively (−) associated with O3I variability, such as age (+), body mass index (−), socioeconomic status (+), and smoking (−) [20]. The O3I correlates well with the tissue levels of Omega-3 fatty acids, as well as the risk of CVD, cognitive dysfunction, depression, and bipolar disorder [21,22]. Indeed, the O3I is independently associated with fatal and non-fatal CVD [23,24], and the risk of these events can be stratified by the category of the O3I (high risk, O3I ≤ 4%; intermediate risk, >4% to <8%; and low risk, ≥8%). Of note, healthy South Korean and Japanese individuals have O3I of 8–12% and 7–11%, respectively, compared to only around 4% for Western populations [25]. Furthermore, a recently published pilot study conducted in the U.S. suggests the O3I may be inversely associated with the risk of death in severe COVID-19 [26]. Thus, the value of the O3I as a blood marker for COVID-19 risk assessment should be thoroughly evaluated. Here, we report the preliminary results of an assessment of the O3I in a cohort of patients with severe COVID-19, as part of an ongoing cross-sectional case-control study.

## 2. Materials and Methods

This study was conducted at two healthcare centers in Rancagua city (Chile) between November 2020 and April 2021. The Hospital Clínico Fusat is a private hospital, and the Hospital Regional Libertador Bernardo O’ Higgins (HRLBO) is the main public hospital in the VI Region of the country and serves as a referral center for a population of around 1 million. The sample size required for a case control study was calculated based on a frequency of exposure of 90% for cases and 70% for controls. The minimum number of cases and controls (equal numbers per group) required to detect an OR of 1.5, with 80% statistical power and 95% security, was 62 individuals. Adult patients admitted to the intensive or intermediate care units with respiratory failure due to COVID-19 were selected to be included in this cross-sectional study. Patient recruitment was largely determined by a recent admission, and informed consent was provided by the patient or their relatives. Sociodemographic and nutritional information, as well as a medical history, were obtained by trained investigators in an interview with the patient (in-person) or their relatives (in person or by phone), and the data were registered using an online questionnaire. Additionally, biometric measures and a drop of blood were obtained directly from the patients after informed consent was provided. The drop of blood was placed on a dry blood spot (DBS) collection card provided by the OmegaQuant^®^ laboratory (Sioux Falls, SD, USA); these DBS collection cards are regularly utilized by the OmegaQuant^®^ laboratory to store and transport samples for O3I determination. According to the laboratory instructions [26], the DBS collection cards were frozen at 22° Celsius immediately after sample collection for a period of time ranging from a few days to 5 months, then shipped to the OmegaQuant^®^ laboratory in the U.S. by regular airmail; the whole process of thawing and shipping the DBS collection cards took 5 days. Importantly, the dried blood spot samples remain stable on the collection cards for up to 30 days at room temperature and 12 months at −22° Celsius. Additionally, blood samples were collected from 10 healthy subjects as quality controls for the process of storing and shipping the DBS collection cards; the majority of these subjects had been taking Omega-3 fatty acid supplements (dose range of EPA + DHA between 1 and 2 g) daily for at least 4 months. The blood samples of the quality-control subjects were obtained at different time points throughout the study period. Additionally, a complete RBC fatty-acid profile analysis was carried out for every sample. Importantly, the OmegaQuant^®^ laboratory (Sioux Falls, SD, USA) remained blinded to the condition of the subjects (patients or quality-control individuals).

The information was collected using an online formulary that generates an Excel spreadsheet. The data analysis was performed in SPSS (V26) IBM Statistics^®^ (Armonk, NY, USA). Standard descriptive analysis (means and standard deviations, medians and ranges, and counts and percentages) are used to present the results. The list of cases was sorted and grouped into four quartiles based on the O3I. To assess the relationships between the O3I and mechanical ventilation and death, the lower and upper quartiles were compared to the other quartiles using the Chi-square Fisher’s exact test. Finally, binary logistic regression models were executed to assess if the O3I quartiles are associated with the requirement for mechanical ventilation and death, adjusting for age, sex, and other factors such as BMI, tobacco consumption, and the three major co-morbidities (diabetes, hypertension and chronic obstructive pulmonary disease). For all tests, statistical significance was defined as a *p*-value lower than 0.05 (two-sided).

The study protocol was approved by the Ethics Committee of the Faculty of Medicine of Universidad Católica del Norte (Resolution number 22/2020).

## 3. Results

A total of 75 patients were recruited, almost all (98%) at the HRLBO. One patient was excluded because the entire RBC fatty-acid profile of their sample showed unequivocal indicators of degradation; this patient had severe comorbidities and died 2 h after the sample was drawn.

The demographic and clinical characteristics of the remaining 74 subjects are shown in Table 1. A substantial proportion of patients were 65 years old or above and had chronic CVD and obesity; mechanical ventilation and several weeks of hospital stay were typically required. The O3I levels of the patients with severe COVID-19 were low. Importantly, the O3I levels of the healthy quality-control subjects were in the intermediate to high range (average: 7.84%; range: 4.65–10.71%). The quality-control subjects (*n* = 10) were an average age of 50.8 years old (range, 36–69), average BMI of 27.4 (range, 23.0–35.3), and 90% were taking Omega-3 supplements.

Table 2 describes the variables of interest in the patient cohort stratified by the O3I quartiles. All variables exhibited similar distributions among the quartiles. The differences observed in the frequency distributions for tobacco smoking and Omega-3 supplementation were not significant. Around 50% of the subjects reported consuming fish less than twice a week, and frying as a common way of cooking fish. Very few patients (*n* = 3) were taking Omega-3 supplements; these patients presented O3I values over 4.16%, corresponding to the higher quartiles (Q3 and Q4).

The associations of the O3I with death and mechanical ventilation are presented in Table 3 and Table 4, respectively, based on comparison of the O3I quartiles using multivariate logistic regression analysis. Subjects in the lowest O3I quartile (<3.57%) had a 3.1-fold higher risk of dying from severe COVID-19 than the patients in the highest quartile (OR 3.311; IC 1.261–7.676; *p* = 0.032). Patients in the highest O3I quartile (>4.52%) had a lower risk of requiring mechanical ventilation, compared to other quartiles (OR 0.257; IC 0.083–0.791; *p* = 0.026). According to the binary logistic regression models, a higher O3I reduced the risk of mechanical ventilation by half (OR 0.48; C.I. 95% [0.233–0.987]; *p* = 0.046). The results of the model adjusted by age and sex were similar (OR 0.469; C.I. 95% [0.227–0.969]; *p* = 0.041) and also when adjusted for other factors, such as the three main co-morbidities (diabetes, hypertension, chronic pulmonary disease), tobacco consumption, and body mass index. A higher O3I also reduced the risk of death; this relationship became significant after adjusting by age and sex (OR 0.299; C.I. 95% [0.092–0.976]; *p* = 0.046) and remained significant when adjusted for other factors of interest. In the last two models, the variable age significantly influenced the risk of death.

## 4. Discussion

We observed an inverse association between the O3I and major clinical endpoints of severe COVID-19, and these associations remained statistically significant after adjusting for well-known risk factors in severe COVID-19. However, more research is needed to clearly establish the O3I as a blood marker for COVID-19 risk assessment. Although few laboratories in the world have implemented the standardized methodology for determination of the O3I, studies on this topic can be conducted worldwide due to the long-term stability of blood samples on DBS collection cards.

The substantial variation in the O3I between patients with severe COVID-19 and the healthy quality-control subjects clearly indicates that O3I reflects the Omega-3 status of these individuals. The specific nutritional information gathered through the dietary survey and the complete RBC fatty-acid profiles of every patient and healthy quality-control individual further support the validity of the O3I results in this study. Blood levels of Omega-3 fatty acids are increasingly included as exposure variables in epidemiologic and clinical studies because the O3I correlates better with morbimortality indices than the reported intake of Omega-3 fatty acids. Furthermore, the O3I should be measured, as it cannot be reliably predicted [18,20].

The patients with severe COVID-19 had low O3I values, which is characteristic of western populations (11) and corresponds well with the patients’ low dietary fish intake and negligible Omega-3 fatty acid supplementation [27]. Additionally, the reported common habit of frying as a means of cooking fish could reduce the bioavailability of the nutrients EPA and DHA [28,29]. The concordance between the distribution of fish consumption and O3I quartiles is not as expected. However, the consumption of supplements seems to be more reliable and effective, in agreement with publications that indicate the consumption of fish alone is insufficient to maintain high O3I values [18,27]. The narrow range of very low values of the measured indicator (O3I), together with the relatively small sample size, could explain the difference found in the proportions of deceased individuals among quartiles and the lack of a relationship between the death rate and mechanical ventilation. We hypothesize that including subjects with a wide range of O3I values will clarify this issue.

Notably, the inverse associations between the O3I with the outcome variables were observed within a relatively narrow range of O3I values, which strongly suggests that COVID-19 risk stratification based on O3I categories may indeed be feasible [26]. This finding also reinforces the need to include the basal O3I of eligible participants in future randomized clinical trials of Omega-3 fatty acids in COVID-19 [30,31,32]; unbiased research requires valid comparisons, and the predictive value of biomarkers should be validated in well-designed prospective clinical studies. Increasing evidence strongly suggests the omega-6:Omega-3 fatty acid ratio should be replaced by a metric that focuses on the primary deficiency in Western diets—the lack of EPA and DHA [33]; namely, the Omega-3 Index.

The outcome and exposure variables are measured at the same time in cross-sectional studies, which limits the ability to draw causal inferences from this type of observational study. However, based on the lifetime of RBCs, determination of the O3I reflects the individuals’ Omega-3 status over the previous 4 months, which would certainly precede the outcomes of an acute infectious disease. Importantly, the quality of an individual’s immune response could be strongly influenced by their Omega-3 status [14,15,34,35], along with other nutrients such as zinc, selenium, and vitamins D, C, and E [36]. Measuring the levels of those nutrients would have provided supplementary information regarding the current nutritional status of patients with severe COVID-19 and low fish intake.

The relatively small sample size, along with the non-randomized recruitment of subjects, may have increased the risk of selection bias, and these factors represent the main methodological limitations of our study.

## 5. Conclusions

Omega-3 status may profoundly influence the homeostasis of inflammation resolution and airway mucosal immunity. The novelty of this study is that it confirms the already reported relationship between the O3I and the clinical outcomes of SARS-CoV-2 infection, together with other clinical and demographic variables and consumption of fish and supplements, using an observational design in a cohort of patients with severe COVID-19 from a non-intervention population. Addressing the widespread Omega-3 fatty-acid deficiency at the population level could have far-reaching implications on the evolution of the COVID-19 pandemic. We present preliminary evidence supporting the hypothesis that the risk of severe COVID-19 can be stratified based on the O3I category. The value of the O3I as a blood marker for COVID-19 risk assessment warrants further investigation.

## Figures and Tables

**Table 1:** Demographic and clinical characteristics of the patients with severe COVID-19 (*n* = 74).

Qualitative Variable	Categories	Number	Percentage
Sex	Male	39	52.7
	Female	35	47.3
Co morbidities *	Hypertension	35	47.3
	Diabetes	27	36.5
	Asthma, chronic lung disease	12	16.2
	Chronic kidney disease	5	6.8
	Heart disease	5	6.8
	Immunosuppressive treatment	2	2.7
	HIV/AIDS	1	1.4
	Cancer	0	0.0
Tobacco consumption		6	8.1
Symptoms of COVID-19	Respiratory distress	70	94.6
	Fatigue	44	59.5
	Cough	37	50.0
	Fever	31	41.9
	Muscular pain	23	31.1
	Headache	8	10.8
	Throat pain	5	6.8
	Chest pain	4	5.4
	Loss of smell	3	4.1
	Loss of taste	3	4.1
	Abdominal pain or diarrhea	2	2.7
Mechanical ventilation required		43	58.1
Death		14	18.9
**Quantitative variable**	**Mean ± Standard deviation**	**Minimum**	**Maximum**
Age	59.68 ± 13.6	21	82
Body Mass Index	29.47 ± 6.14	20	44.5
Oximetry	87% ± 7.2%	58.00%	98.00%
Days of hospital stay	21.5 ± 12.39	3	68
Omega-3 Index	4.15% ± 0.69%	3.06%	6.14%

(*) One patient may have more than one morbidity.

**Table 2:** Demographic, clinical, and fish consumption profiles of the participants stratified by O3 Index quartiles.

Categorical by O3 Index Quartile		Q1: ≤3.56% (*n* = 19) [*n* (%)]	Q2: 3.57–4.15% (*n* = 18) [*n* (%)]	Q3: 4.16–4.52% (*n* = 19) [*n* (%)]	Q4: ≥4.53% (*n* = 18) [*n* (%)]	Total Mean ± SD [*n* (%)]
Age	(Mean ± SD)	60.3 ± 11.9	57.6 ± 11.1	60.0 ± 15.5	60.8 ± 15.9	59.68 ± 13.6
Sex	(Male)	12 (63.2)	10 (55.6)	7 (36.8)	10 (55.6)	39 (52.7)
BMI	(Mean ± S.D.)	26.6 ± 5.5	30.8 ± 5.9	30.9 ± 6.5	29.5 ± 6.3	29.47 ± 6.14
Tobacco consumption		4 (21.1)	1 (5.6)	1 (5.3)	0.0	6 (8.1)
Diabetes		5 (26.3)	9 (50.0)	6 (31.6)	7 (38.9)	27 (36.5)
Hypertension		6 (31.6)	9 (50.0)	10 (52.6)	10 (55.6)	35 (47.3)
Asthma or chronic lung disease		2 (10.5)	1 (5.6)	3 (15.8)	6 (33.3)	12 (16.2)
Fish consumption						
	Two or more times a week	4 (21.0)	3 (16.7)	3 (15.8)	2 (11.1)	13 (17.6)
	Less than two times a week	13 (63.2)	10 (55.6)	14 (63.2)	13 (72.2)	50 (67.5)
	Does not consume	2 (10.5)	4 (22.2)	2 (10.5)	3 (16.7)	11 (14.9)
Type of fish consumed						
	Salmon, mackerel, saw (Over 300 mg O3/100 g)	4 (21.1)	6 (33.3)	3 (15.8)	5 (27.8)	18 (24.3)
	Tuna, hake, croaker, pippin (200–300 mg O3/100 g)	13 (68.4)	8 (44.4)	14 (73.7)	10 (55.6)	45 (60.1)
	Does not consume	2 (10.5)	4 (22.2)	2 (10.5)	3 (16.7)	11 (14.9)
Method of cooking fish						
	Oven, griddle, pot, canned, raw	10 (52.7)	5 (27.8)	10 (52.7)	6 (33.3)	31 (41.9)
	Fried	7 (36.8)	9 (50.0)	7 (36.9)	9 (50.0)	32 (43.2)
	Does not consume	2 (10.5)	4 (22.2)	2 (10.5)	3 (16.7)	11 (14.9)
O3 supplement consumption		0.0	0.0	1 (5.3)	2 (11.1)	3 (4.1%)
Mechanical ventilation		14 (73.7)	12 (66.6)	11 (57.9)	6 (33.3)	43 (58.1)
Death		7 (36.8)	1 (5.6)	5 (26.3)	1 (5.6)	14 (18.9)

O3: Omega-3; Q: Quartile; BMI: Body Mass Index; SD: Standard deviation; *n*: Number of individuals.

**Table 3:** Risk of mechanical ventilation and death among the highest and lowest O3I quartiles compared to other quartiles of patients with severe COVID-19.

	OR	C.I.	*p* *
Risk of MV for the lowest O3I quartile (<3.57%) compared to higher quartiles	1.348	0.925–1.964	0.183
Risk of death for the lowest O3I quartile (<3.57%) compared to higher quartiles	3.111	1.261–7.676	0.032
Reduction in the risk of MV for the highest O3I quartile (>4.51%) compared to the lowest quartile	0.257	0.083–0.791	0.026
Reduction in the risk of death for the highest O3I quartile of (>4.51%) compared to the lowest quartile	0.195	0.024–1.605	0.165

OR: Odds ratio; C.I.: Confidence interval; MV: mechanical ventilation; O3I: Omega-3 Index; *p* *: *p*-value of Chi^2^ Fisher’s exact test (two-sided).

**Table 4:** Model of the associations of the Omega-3 Index with mechanical ventilation and death in the patients with severe COVID-19.

Clinical Outcome	Unadjusted Model			Adjusted by Age and Sex				Adjusted by Age, Sex, Comorbidities, BMI and Tobacco Use			
	OR	C.I.	*p*		OR	C.I.	*p*		OR	C.I.	*p*
Mechanical	0.48	0.233–0.987	0.046	Full model	0.469	0.227–0.969	0.041	Full model	0.459	0.211–0.997	0.049
ventilation				Age (years)	0.993	0.958–1.029	0.680	Age (years)	0.998	0.957–1.042	0.945
				Sex (male)	2.206	0.834–5.836	0.111	Sex (male)	2.327	0.834–6.493	0.107
								Diabetes	1.061	0.340–3.314	0.919
								Hypertension	0.599	0.183–1.966	0.398
								COPD	1.179	0.272–5.106	0.826
								BMI	0.993	0.910–1.084	0.881
								Tobacco	1.252	0.152–10.330	0.834
Death	0.37	0.128–1.071	0.067	Full model	0.299	0.092–0.976	0.046	Full model	0.28	0.08–0.985	0.047
				Age (years)	1.070	1.009–1.138	0.025	Age (years)	1.083	1.013–1.158	0.019
				Sex (male)	1.689	0.466–6.129	0.425	Sex (male)	1.842	0.453–7.496	0.394
								Diabetes	1.367	0.306–6.104	0.682
								Hypertension	0.408	0.09–1.482	0.244
								COPD	2.388	0.391–14.599	0.346
								BMI	1.044	0.931–1.172	0.462
								Tobacco	0.883	0.063–12.282	0.412

OR: Odds ratio; C.I.: Confidence interval; COPD: Chronic obstructive pulmonary disease; BMI: Body mass index; *p*: *p*-value.

## Data Availability

Laboratory results data can be requested from the corresponding author, excluding identification and clinical data of the patients.
